# Effect of tranexamic acid administration on acute traumatic coagulopathy in rats with polytrauma and hemorrhage

**DOI:** 10.1371/journal.pone.0223406

**Published:** 2019-10-03

**Authors:** Xiaowu Wu, Avi Benov, Daniel N. Darlington, Jeffrey D. Keesee, Bin Liu, Andrew P. Cap

**Affiliations:** 1 Coagulation and Blood Research Program, United States Army Institute of Surgical Research, Fort Sam Houston, Texas, United States of America; 2 Department of Surgery “A”, Meir Medical Center, Kfar Saba and the Sackler School of Medicine, Tel-Aviv University, Tel-Aviv, Israel; Medical College of Georgia, Augusta, UNITED STATES

## Abstract

Trauma and hemorrhagic shock can lead to acute traumatic coagulopathy (ATC) that is not fully reversed by prehospital resuscitation as simulated with a limited volume of fresh whole blood (FWB) in a rat model. Tranexamic Acid (TXA) is used as an anti-fibrinolytic agent to reduce surgical bleeding if administered prior to or during surgery, and to improve survival in trauma if given early after trauma. It is not clear from the existing clinical literature whether TXA has the same mechanism of action in both settings. This study sought to explore the molecular mechanisms of TXA activity in trauma and determine whether administration of TXA as a supplement to FWB resuscitation could attenuate the established ATC in a rat model simulating prehospital resuscitation of polytrauma and hemorrhagic shock. In a parallel in-vitro study, the effects on clotting assays of adding plasmin at varying doses along with either simultaneous addition of TXA or pre-incubation with TXA were measured, and the results suggested that maximum anti-fibrinolytic effect of TXA on plasmin-induced fibrinolysis required pre-incubation of TXA and plasmin prior to clot initiation. In the rat model, ATC was induced by polytrauma followed by 40% hemorrhage. One hour after trauma, the rats were resuscitated with FWB collected from donor rats. Vehicle or TXA (10mg/kg) was given as bolus either before trauma (TXA-BT), or 45min after trauma prior to resuscitation (TXA-AT). The TXA-BT group was included to contrast the coagulation effects of TXA when used as it is in elective surgery vs. what is actually feasible in real trauma patients (TXA-AT group). A single dose of TXA prior to trauma significantly delayed the onset of ATC from 30min to 120min after trauma as measured by a rise in prothrombin time (PT). The plasma d-dimer as well as plasminogen/fibrinogen ratio in traumatized liver of TXA-BT were significantly lower as compared to vehicle and TXA-AT. Wet/dry weight ratio and leukocytes infiltration of lungs were significantly decreased only if TXA was administrated later, prior to resuscitation (TXA-AT). In conclusion: Limited prehospital trauma resuscitation that includes FWB and TXA may not correct established systemic ATC, but rather may improve overall outcomes of resuscitation by attenuation of acute lung injury. By contrast, TXA given prior to trauma reduced levels of fibrinolysis at the site of tissue injury and circulatory d-dimer, and delayed development of coagulopathy independent of reduction of fibrinogen levels following trauma. These findings highlight the importance of early administration of TXA in trauma, and suggest that further optimization of dosing protocols in trauma to exploit TXA’s various sites and modes of action may further improve patient outcomes.

## Introduction

Trauma induced hemorrhage is a leading cause of mortality and morbidity in both civilian and military casualties [1, 2]. Acute traumatic coagulopathy (ATC) develops in one third of trauma patients [3, 4], leading to increased bleeding and more frequent massive transfusion. Therefore, trauma patients with ATC have higher mortality rates in comparison to those without ATC. Hemostatic damage control resuscitation is currently implemented in trauma management in order to restore not only the hemodynamic but also the hemostatic deficit. Fresh whole blood (FWB) restores hemodynamic deficits in hemorrhagic shock while minimizing dilution effects that potentially contribute to ATC. Fresh whole blood (FWB) is associated with superior outcomes compared to component therapy in the treatment of trauma with hemorrhagic shock [5, 6].

Recent evidence in both animal and clinical studies has shown that ATC has a fibrinolytic component. Trauma patients show an elevation in plasma d-dimers [7, 8], tissue plasminogen activator (tPA) and plasmin-antiplasmin complex (PAP) [9, 10]. Rats subjected to severe trauma develop an elevation over time in d-dimers, tPA, and plasmin activity [11], with a subsequent fall in clot strength (12). The activation of fibrinolysis and the loss of clot strength suggest mechanisms that would explain why anti-fibrinolytic drugs have demonstrated benefit in the treatment of trauma [13–16].

Tranexamic acid (TXA) is a lysine derivative that exerts anti-fibrinolytic activity by blocking the lysine binding site of plasminogen or plasmin [17]. TXA reduces activation of plasmin from plasminogen by preventing plasminogen from associating with tPA on the surface of fibrin strands. TXA administration has been demonstrated to reduce blood loss in patients undergoing major surgery and in postpartum hemorrhage [18, 19] when administered at the beginning of or during surgery, suggesting that the principle mechanism of TXA is associated with reduction of fibrinolysis and improvement of clot stability at the site of tissue injury. Clinical studies have shown that TXA administration improves survival rates for trauma patients, especially those with active bleeding (CRASH-2) [13, 20]. This beneficial effect has also been demonstrated in studies among military casualties [14, 15]. Clinical data from prophylactic use in elective surgery and the CRASH-2 trial in trauma suggest that timing of administration in relation to coagulation and immune system activation caused by either surgery or trauma may determine different biological outcomes. It is not clear from the existing clinical literature whether TXA has the same mechanism of action in both settings of surgery and trauma. An improved understanding of the effects of TXA on the complex physiology of ATC is clearly needed.

Plasmin causes not only fibrinolysis but also up-regulation of inflammatory responses and activation of bradykinin signaling which leads to damaged vascular integrity and increased vascular permeability [21]. Based on our previous study showing decreased tissue neutrophil infiltration, complement activation, plasmin activity and lung wet/dry weight ratio following TXA administration [22], we currently sought to correlate potential changes in coagulation parameters with changes in markers of immune mediated tissue injury. The possible correlation of immune mediated tissue injury with readily obtained coagulation parameters could be clinically useful.

In a rat model with ATC induced by polytrauma and hemorrhage [12], we previously demonstrated that limited FWB resuscitation (designed to model prehospital blood transfusion) restored hemodynamic deficits, but had little impact on coagulopathy as characterized by prolongation of prothrombin time (PT). This study aims to explore the molecular mechanisms of TXA activity in trauma and determine whether administration of TXA as a supplement to FWB resuscitation could attenuate development of ATC using a rat model simulated advanced military and civilian prehospital resuscitation protocols for polytrauma and hemorrhagic shock. In particular, this investigation also explored the localization and kinetics of plasminogen to plasmin conversion upon tissue injury and the relation of these parameters to other indices of coagulation function by comparing the effects of TXA administration prior to and after trauma We hypothesize that, 1) giving TXA prior to coagulation/immune activation (pre-injury) would conceptually result in greatest improvement in coagulation parameters (fibrinolysis and clot stability) compared to administration after trauma when plasminogen would have had a chance to incorporate into forming clots; and 2) TXA administration after trauma leads to attenuation of trauma associated organ immune injury.

## Materials and methods

The animal study was approved by the Institutional Animal Care and Use Committee of the U.S. Army Institute of Surgical Research, and conducted in compliance with the Animal Welfare Act, the implementing Animal Welfare Regulations, and the principles of the “Guide for the Care and Use of Laboratory Animals”. All experiments were started between 0800–0900 hours in a room separated from home caging. All rats (Sprague-Dawley from Charles Rivers, www.criver.com) were male, and were group housed before the experiment. The light/dark cycle was 12 hours light/12 hours dark. The rats ate Laboratory Rodent Diet 5001 (www.LabDiet.com). Food and water were given ad libitum. Thirty rats were randomly and evenly assigned to three study groups: vehicle, TXA given before trauma (TXA-BT), and TXA given after trauma (TXA-AT). Our power analysis determined that a significant difference (alpha of 0.05 with a power of 0.80) in prolongation of PT (difference in means: 2.7 and 2.5 seconds, and standard deviation: 1.5 and 1.4 respectively) could be detected with groups of 6 to 7 rats at 2hr after trauma without or with FWB resuscitation [12, 23].

In a parallel in-vitro study, coagulation properties were measured in human whole blood (n = 4) treated with plasmin, TXA or plasmin/TXA. This study was conducted under a protocol reviewed and approved by the US Army Medical Research and Materiel Command Institutional Review Board and in accordance with the approved protocol. Human whole blood was collected from healthy volunteers with both verbal and written consent.

Coagulation properties in-vitro: human whole blood was collected in 3.8% sodium citrate. The whole blood was treated with either plasmin (250, 100, 25μg/ml, Millipore Sigma, St. Louis, MO), TXA (400μg/ml, equivalent to current in vivo therapeutic dose (10mg/kg, Cyklokapron^®^)), or plasmin with TXA. For combination treatment of plasmin and TXA, the plasmin and TXA were either pre-incubated for 10min (TXA-PI) or added simultaneously (TXA-S) immediately prior to assay. Whole blood without any treatment was used as a baseline (control) for all groups. Prothrombin time (PT), and activated partial thromboplastin time (aPTT) was measured by Start-4 (Diagnostic Stago Inc, Parsippany, NJ). Coagulation Time (CT), Maximum Clot Firmness (MCF) and Lysis Index (LI) were measured by rotational thromboelastography (ROTEM, Munich, Germany).

Polytrauma with hemorrhage: We used a well-characterized rat model that is described in detail elsewhere [11, 12]. Briefly, Sprague-Dawley rats (350-400g) were anesthetized with 1–2% isoflurane/100% oxygen through a nose cone and allowed to breathe spontaneously. The amount of isoflurane needed will be assessed by noting a lack of response to a toe pinch and monitoring arterial pressure and heart rate. The left femoral artery and vein were cannulated for monitoring arterial blood pressure, and blood withdrawal/transfusion respectively. Polytrauma was started within 10min from cannulation by laparotomy, followed by crush injury to left and medial liver lobes (3 crushes to each lobe), and 10cm section of small intestines at proximal end of the cecum (10 separate crushes). After the abdominal incision was closed, a closed fracture of the right femur was made using a modification of a 3-point impact device by dropping six 65g stainless steel balls from 36” through a guide tube, followed by crushing right leg skeletal muscle for 10 times. The bleeding caused by tissue injury was estimated less than 1ml in total and was not counted into total volume of hemorrhage. The trauma procedures were completed within 20 min after which the post-trauma period began. The rats were then bled to a mean arterial pressure of 40 mmHg within 5min and maintained at 40mmHg until 40% of estimated blood volume was removed. Blood volume was estimated as 7% of body weight. Hemorrhage was completed between 30 and 45 min after trauma, but varied as each rat compensated differently. Hemorrhage was then discontinued, and blood pressure and heart rate were allowed to freely compensate. At 2hr after trauma, rats were euthanized via exsanguination.

Resuscitation with FWB and administration of TXA: Rats were resuscitated 60min after completion of trauma with FWB collected from donor rats. Donors were anesthetized and cannulated as above, then exsanguinated under anesthesia. FWB was collected in CPD (citrate phosphate dextrose) and was ready for transfusion within 1hr from collection (20% of blood volume or 50% of shed volume was used for resuscitation, which is equivalent to giving 2 units of whole blood to a 70kg human). Tranexamic acid (TXA, 10mg/kg, Cyklokapron^®^) was given intravenously as a bolus (diluted in 200μl with normal saline) before trauma (TXA-BT, after the blood sample drawn for baseline, and immediately before trauma procedures) or 45min after trauma (TXA-AT, 15min prior to resuscitation with FWB). The dose of TXA was determined from the CRASH-2 study using 1g TXA bolus administration for trauma patients (10-15mg/kg bolus based on a subject with 70-100kg body weight) [20]. This study modeled the prehospital resuscitation of trauma and hemorrhage, where the additional I.V. infusion dose of TXA for 8hr (in the CRASH-2 study) is impractical and rarely given by prehospital personnel so it was not applied in this study. The control rats received the same polytrauma and hemorrhage and FWB resuscitation as TXA-treated rats, but were given a similar volume of normal saline prior to trauma instead of TXA. All rats were euthanized one hour after resuscitation. The blood samples were taken at baseline (prior to TXA-BT and trauma), 30min and 120min from completion of trauma procedure. Complete blood cell count was measured by Coulter Ac Tdiff2 (Beckman Coulter, Brea, CA). PT, aPTT, and fibrinogen concentration were measured by Start-4 (Diagnostic Stago Inc, Parsippany, NJ).

Tissue collection and procession: The lung and traumatized liver were harvested at the end of the experiment. The right inferior lobe of the right lung was taken for wet weight, and then kept in a drying oven at 60 ^0^C for 10–14 days until the weight (as dry weight) had not changed for three consecutive days. The wet/dry weight ratio was calculated, representing the lung water content as an indicator of lung injury. The left lobe of lung and the liver tissue (included the site of injury) were processed for immunohistochemistry as described in detail below.

Plasmin activity and D-dimer in the plasma: Plasmin activity was measured by chromogenic substrate (S-2403, Chromogenix, DiaPharma Group, Inc., West Chester, Ohio). Prior to adding the chromogenic substrate, both plasmin standard and samples were treated with hirudin (25mg/ml, HYPHEN BioMed, Neuville-Sur-Oise, France) to avoid cross reaction with thrombin. Human plasmin (Millipore Sigma, St. Louis, MO) was used to generate the standard curve by measuring the absorbance change over time at 405nm by a spectrophotometer (BioTek Instruments, Inc, Winooski, VT). The change of absorbance over time in the samples was then measured and the plasmin activity was determined by standard curve. D-dimer was measured by ELISA kit (MyBioSource, WuHan, China) following manufacture’s instruction.

Immunohistochemistry: The liver and lung tissues were fixed in 4% paraformaldehyde overnight, followed by 20% sucrose for 24 hours. Following fixation, the tissues were then embedded in Optimal Cutting Temperature Compound (Tissue-Tek, Sakura Finetek USA, Torrance, CA) and frozen in liquid nitrogen for cryosectioning at 5μm thickness respectively. The liver sections were treated with goat polyclonal anti-rat plasminogen (Abcam, Cambridge, UK) and rabbit polyclonal anti-rat fibrinogen (Abcam, Cambridge, UK), followed by treatment with secondary Alexa 594 conjugated anti-goat IgG, and Alexa 488 conjugated anti-rabbit IgG (ThermoFisher Scientific, Grand Island, NY) respectively. Both anti-fibrinogen and anti-plasminogen antibodies did not specifically differentiate fibrinogen and fibrin or plasminogen and plasmin so that the positive stain in the section of traumatized liver tissue represented the amount of fibrinogen or fibrin (fibrin(ogen)) and plasminogen or plasmin (plasmin(ogen)) in the clot respectively. Since there is little free fibrinogen left in the clot, the positive stain of anti-fibrinogen antibody was taken to approximate fibrin content in the clot. The lung sections were treated with goat polyclonal anti-rat plasminogen and mouse anti-rat CD11b (BD Biosciences, San Jose, CA), followed by treatment with secondary Alexa 594 conjugated anti-goat IgG, and Alexa 488 conjugated anti-mouse IgG (ThermoFisher Scientific, Grand Island, NY) respectively. All sections were completed with co-stained with 4',6-diamidino-2-phenylindole (DAPI) for nuclear staining and analyzed under inverted fluorescence microscopy (Zeiss, Oberkochen, Germany). The plasmin(ogen) and fibrin(ogen) stain at the injured area of the liver sections were quantified using ImageJ software (National Institute of Health (NIH), Rockville, MD) to calculate the plasmin(ogen)/fibrin(ogen) ratio, which approximates the proportional amount of plasminogen binding to the fibrin and thus suggests the degree of or potential for fibrinolysis in the tissue. The ratio of plasmin(ogen)/DAPI and CD11b/DAPI in the lung were also quantified by ImageJ to present the leukocytes infiltration, and the co-localization of plasminogen/CD11b was also observed.

**Fig 1:**
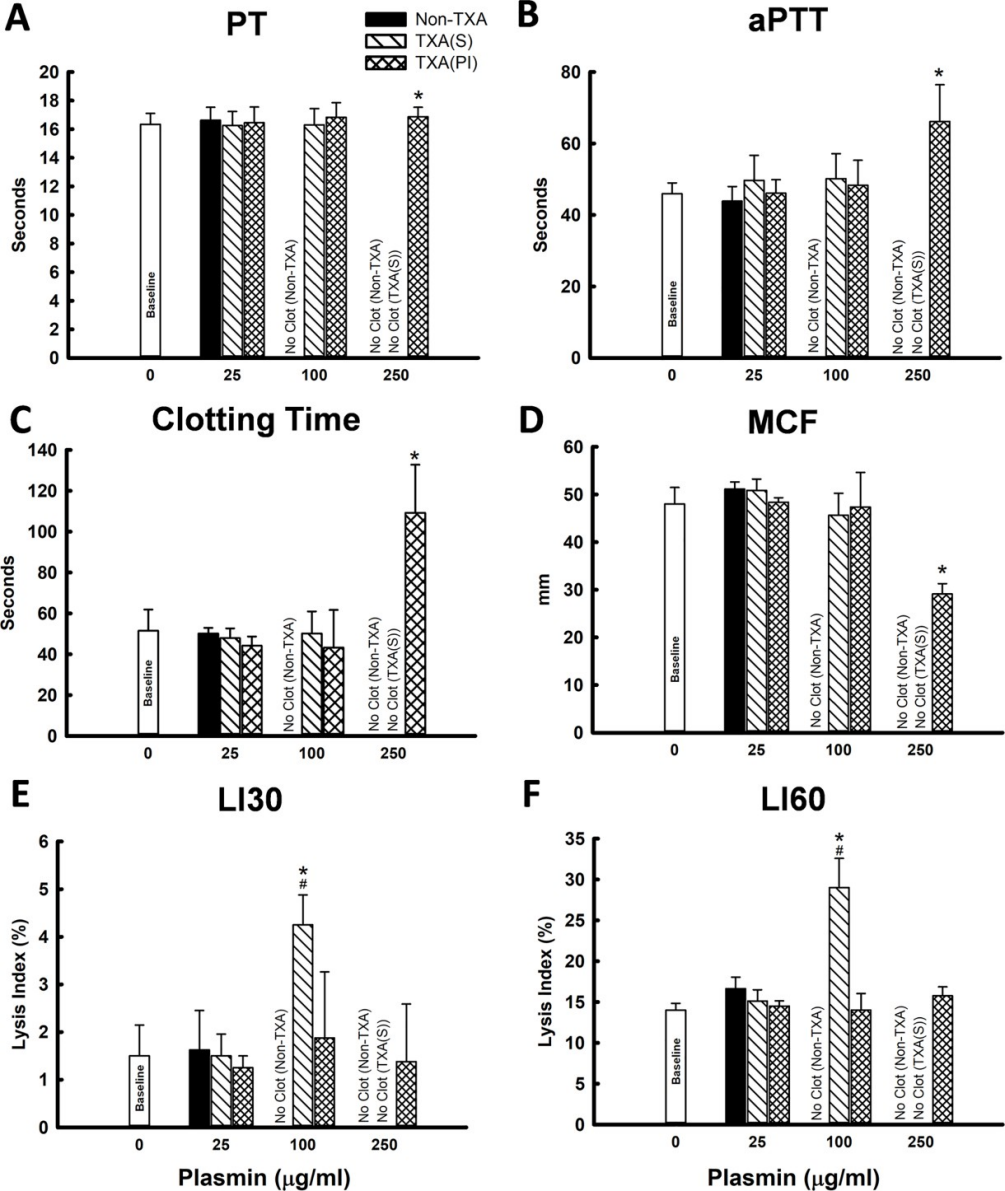
In-vitro assay in human whole blood (n = 4 per group). (A) PT; (B) aPTT; (C) Clotting Time (CT); (D) Maximum Clot Firmness (MCF); (E) Lysis Index at 30min (LI30, the percent reduction in MCF at 30min); (F) Lysis Index at 60min (LI60, the percent reduction in MCF at 60min). Non-TXA: plasmin was added alone when assay started; TXA-S: TXA and plasmin were added simultaneously when assay started; TXA-PI: TXA and plasmin were pre-incubated for 10min before assay started. Baseline: whole blood without treatment of TXA and plasmin. *: significant difference compared to baseline; #: significant difference compared to TXA-PI at given dose of plasmin. (“No Clot” was plotted in the graphs indicating one of following conditions: 1): PT or aPTT were beyond the range of assay (PT >70 seconds; aPTT >120 seconds); 2): CT were >1000 seconds; 3): No reading on MCF and Lysis Index).

**Fig 2:**
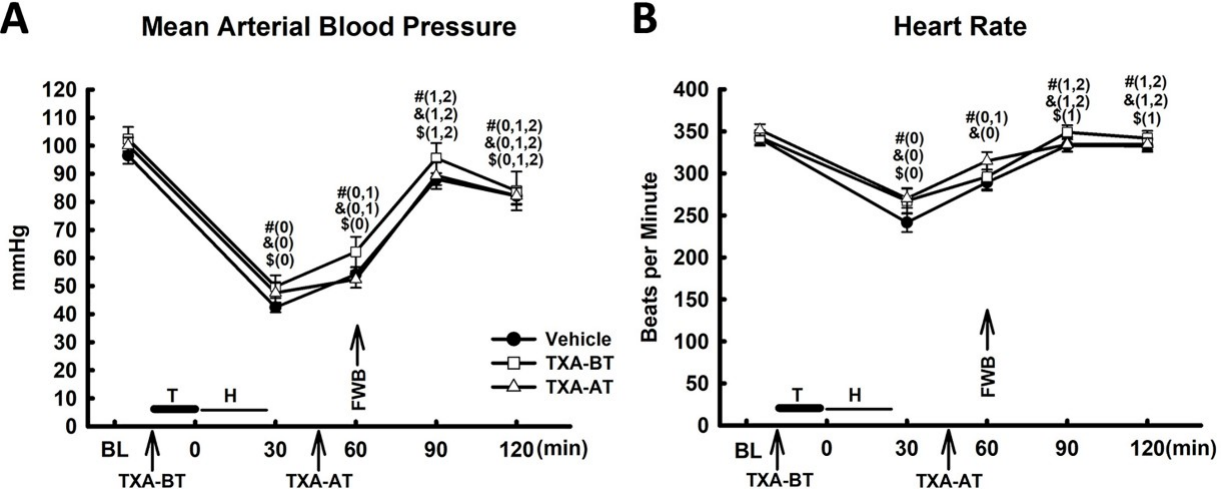
Hemodynamic change (n = 10 per group). (A) Mean arterial blood pressure (MAP); (B) Heart rate. T: Trauma; H: Hemorrhage; FWB: Fresh Whole Blood; #: Vehicle; &: TXA-BT; $: TXA-AT; “0”, “1”, “2” and “3” were presented as a significant difference in comparison to “Baseline”, “30min”, “60min”, and “90min” respectively within the group of Vehicle, TXA-BT or TXA-AT. There was no significant difference in MAP and heart rate among the groups at any given time point.

**Fig 3:**
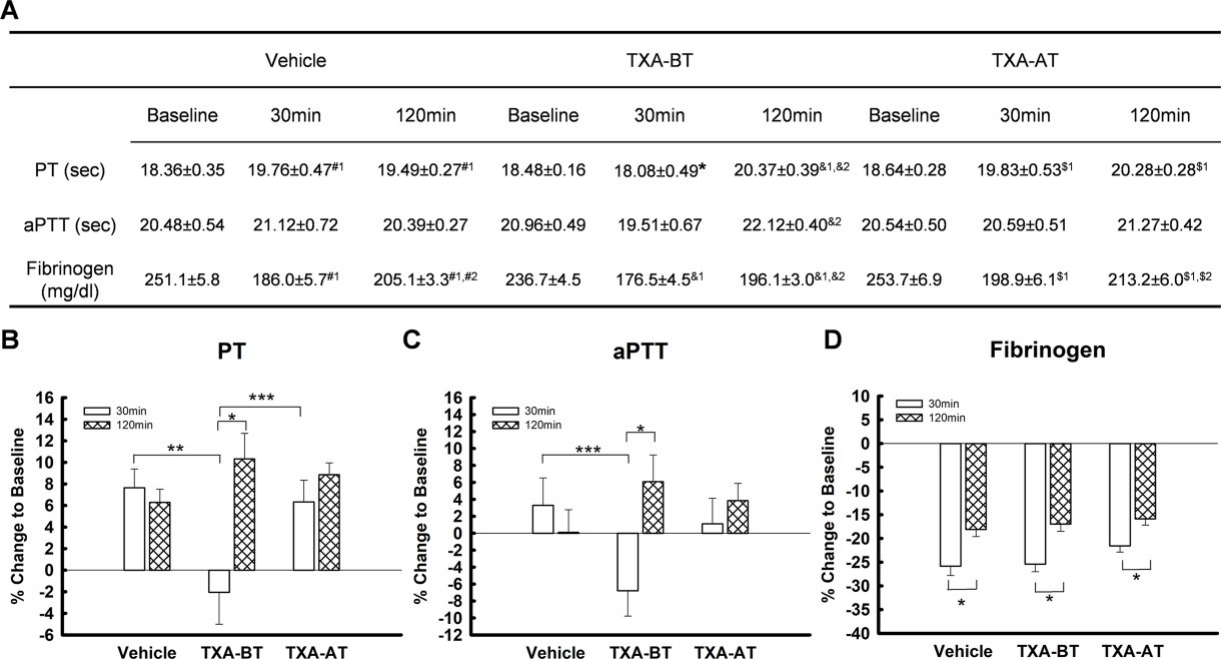
Procoagulant activity (n = 10 per group). (A) Prothrombin time (PT); Activated partial thromboplastic time (aPTT); Fibrinogen. #: Vehicle; &: TXA-BT; $: TXA-AT. #_1_, &_1_ and $_1_: significant difference compared to baseline (BL); #_2_, &_2_ and $_2:_ significant difference compared to 30min. *: significant difference compared to Vehicle and TXA-AT. (B) to (D): percent change to baseline ((B) PT; (C) aPTT; (D) fibrinogen). *: significant difference between 30min and 120min; **: significant difference between vehicle and TXA-BT; ***: significant difference between TXA-BT and TXA-AT. PT and PT% of TXA-BT was significantly lower than that of Vehicle and TXA-AT at 30min.

**Fig 4:**
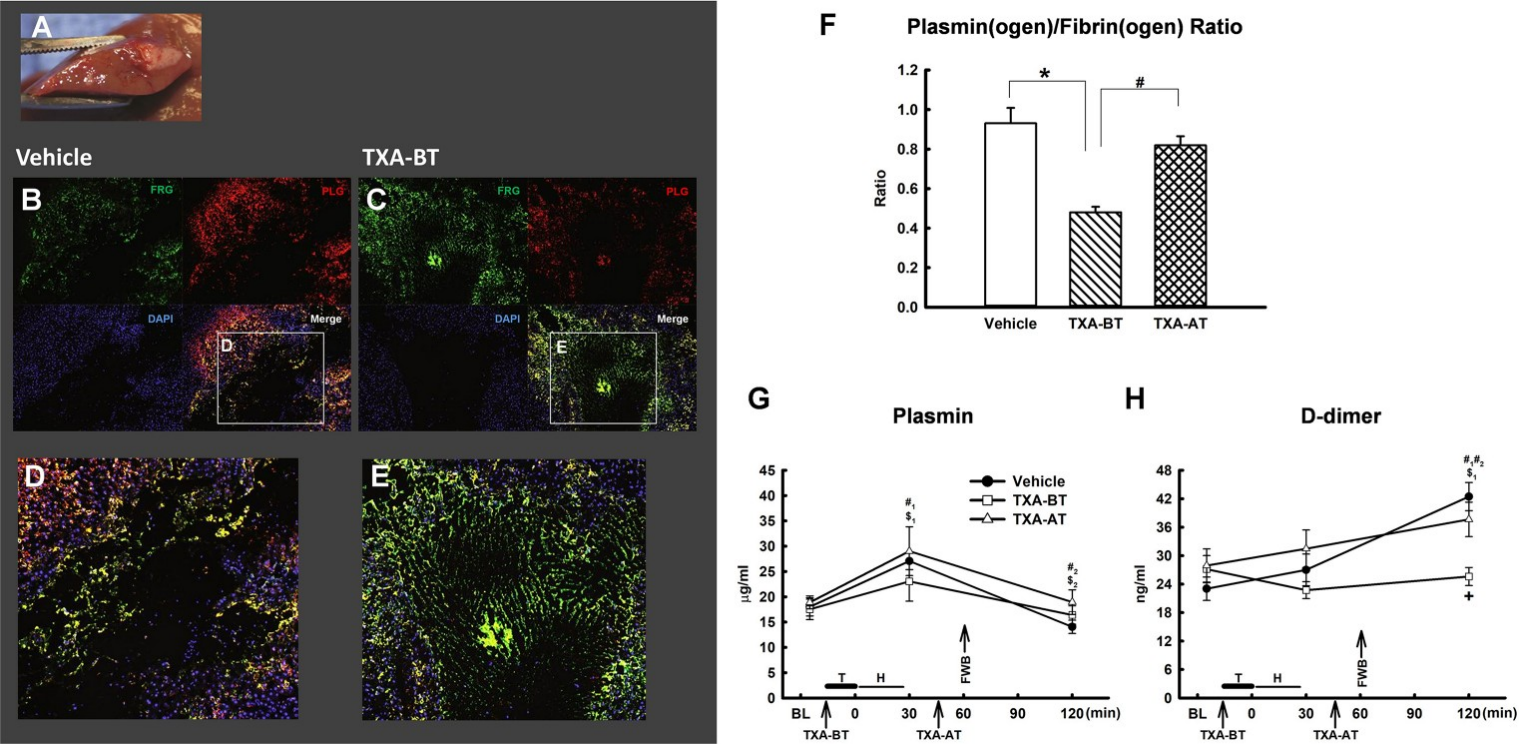
Plasmin(ogen) and fibrin(ogen) at traumatized liver and fibrinolytic activity. (A) Cross section of traumatized liver; (B) and (D) plasminogen and fibrin staining at the injured site of liver of Vehicle; (C) and (E) plasminogen and fibrin staining at the injured site of liver of TXA-BT; (F) Quantification of plasminogen/fibrin ratio at the injured site of liver (n = 8 per group). *: significant difference compared to Vehicle; #: significant difference compared to TXA-AT. (G) and (H) Fibrinolytic Activity (n = 10 per group): (G) Plasmin activity in the plasma; (H) D-dimer levels in the plasma. T: Trauma; H: Hemorrhage; FWB: Fresh Whole Blood. #: Vehicle; &: TXA-BT; $: TXA-AT. #_1_, &_1_ and $_1_: significant difference compared to baseline (BL); #_2_, &_2_ and $_2:_ significant difference compared to 30min.

**Fig 5:**
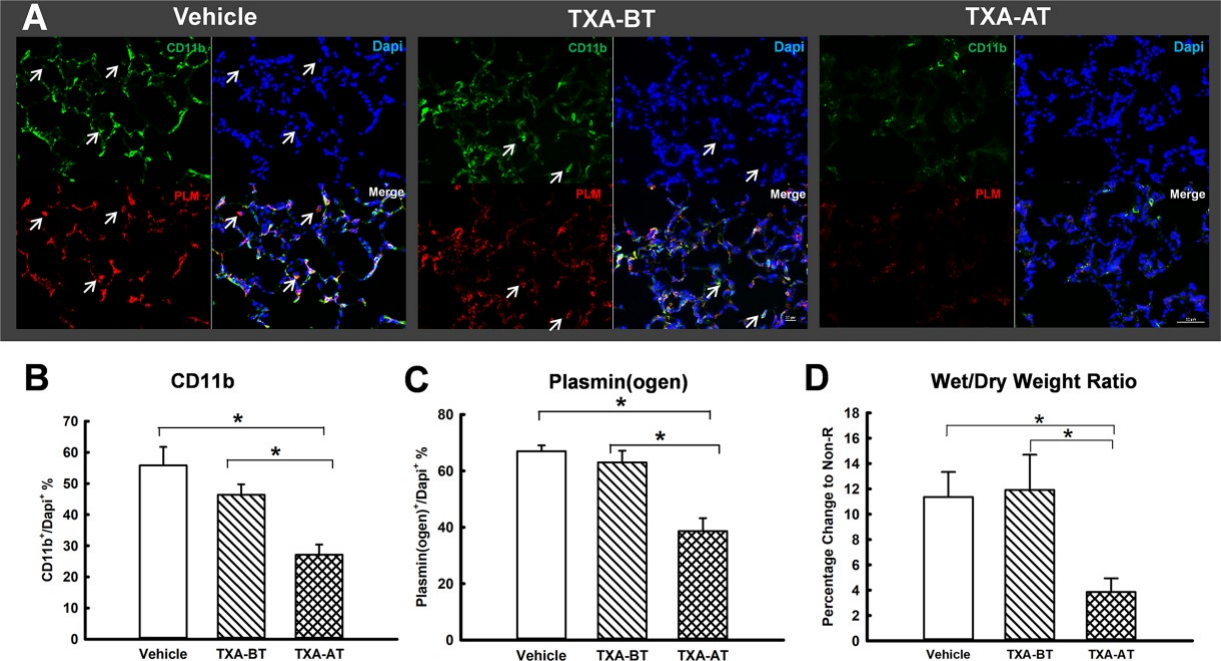
Acute lung injury. (A) Immunohistochemistry staining of CD11b and plasminogen (n = 6 per group); (B) CD11b^+^%; (C) plasmin(ogen)^+^%: * ((B) and (C)): significant difference compared to Vehicle and TXA-BT; arrow sign of (A) indicates cell in the alveoli with co-localized stain of CD11b, plasmin(ogen) (PLM) and DAPI. (D) Lung wet/dry weight ratio (n = 10 per group). The dot line was presented as the range of lung wet/dry ratio from the rats without resuscitation [22]. *: significant difference compared to Vehicle and TXA-BT.

**Fig 6:**
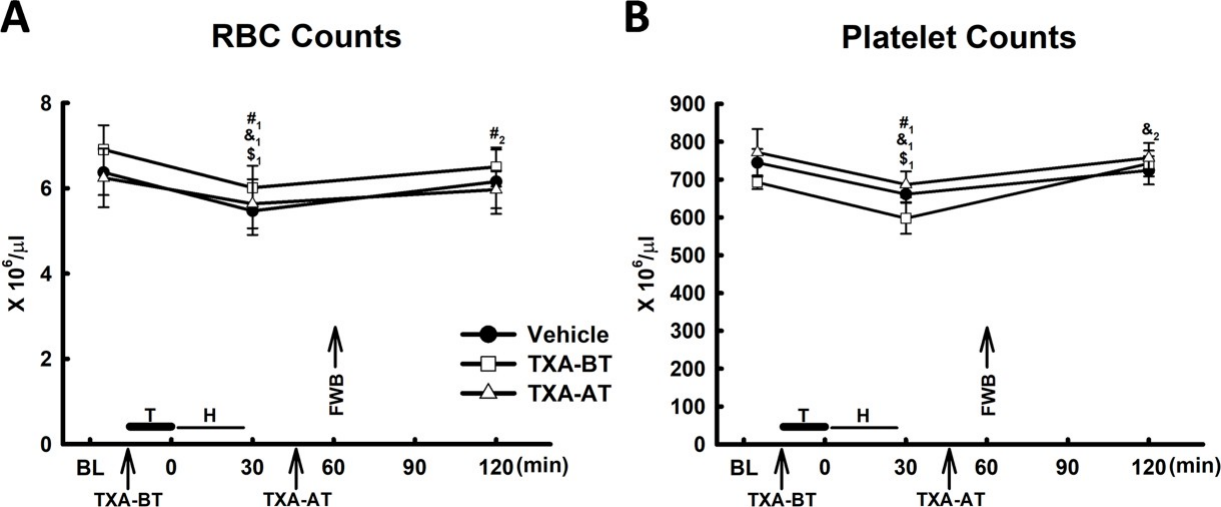
RBC and platelet counts (n = 10 per group). T: Trauma; H: Hemorrhage; FWB: Fresh Whole Blood; A: RBC counts; B: Platelet Counts. #: Vehicle; &: TXA-BT; $: TXA-AT. #_1_, &_1_ and $_1_: significant difference compared to baseline (BL); #_2_, &_2_ and $_2:_ significant difference compared to 30min.

Data analysis: The data analysis was performed by SigmaPlot (Systat Software, Inc. San Jose, CA). All variables were tested for normality using the Shapiro-Wilk test. For in vitro study (Fig 1A–1F) and in vivo study (Figs 2–6), the one-way repeated ANOVA (parametric) or Friedman test (non-parametric) was used to test mean difference among the groups of Control, Non-TXA, TXA(S) and TXA(PI) at each given concentration of plasmin or for the continuous variables over study time points within each group (Vehicle, TXA-BT or TXA-AT) followed by a pairwise comparison with a Tukey method if applicable. Any group that was beyond the range of assay was not included for statistical analysis, and pair t test was used for comparison between two groups. One-way ANOVA or Kruskal-Wallis was used to test mean difference among three treatment groups (Vehicle, TXA-BT or TXA-AT) at each given time point followed by a pairwise comparison with a Tukey method if applicable. Data were presented as means ± SEM (standard error of mean) and statistical significance was accepted at the p<0.05 (2-sided).

## Results

In-vitro assay in human whole blood: Plasmin at 250μg/ml, and 100μg/ml alone completely inhibited coagulation (PT, aPTT exceeded the range of the assay, suggesting no clot formation measured by ROTEM, Fig 1). Plasmin at a dose of 25μg/ml did not affect clot properties as measured by PT, aPTT and ROTEM (CT, MCF, LI30, and LI60) as compared to baseline (Fig 1), and there was no significant difference in PT, aPTT and ROTEM (CT, MCF, LI30, and LI60) among the groups of non-TXA, TXA(S) and TXA(PI). Plasmin at 100 and 250μg/ml completely blocked the clot initiation as measured by ROTEM due to expedited fibrinolysis. Pre-incubation of TXA with plasmin (250μg/ml) reversed the inhibitory effect of plasmin on clot initiation as compared to plasmin alone (non-TXA) and to plasmin with simultaneously added TXA at the start of assay (TXA-S). However, clotting time (CT) was significantly elongated and maximum clot firmness (MCF) was significantly reduced in comparison to baseline (Fig 1C and 1D). Pre-incubation of TXA and plasmin (100μg/ml) completely abolished the effect of plasmin on fibrinolysis by reversal of lysis index at 30min and 60min (LI30 and LI60) as compared to TXA-S (Fig 1E and 1F), which was not significant different to that of control, but there was no significant difference in CT, aPTT, CT and MCF between TXA(S) and TXA(PI) The results suggested that the maximum inhibitory effect of TXA on plasmin-induced fibrinolysis required competitive binding of TXA to plasmin and/or plasminogen prior to fibrin formation, and plasmin at higher dose may affect the clot initiation when the plasmin-induced fibrinolysis exceeded the speed of clot formation.

Hemodynamic change: Polytrauma and hemorrhagic shock led to a significant decline in mean arterial blood pressure (MAP) and heart rate (HR) (Fig 2A and 2B) at 30min and 60min after trauma. Limited resuscitation (20% of blood volume or 50% of shed volume, similar to a 2-unit transfusion in an adult human patient) with FWB at 60min restored both MAP and HR to levels close to baseline at 90min (30min after resuscitation) after trauma. MAP was slightly declined at 120min after trauma, which was significantly lower than MAP at baseline in all three groups. However, there was no significant difference in MAP or HP among the groups at any given time point after trauma and resuscitation, suggesting that TXA given either prior to (TXA-BT) or after trauma (TXA-AT) had no significant impact on the change of MAP or HR in this model.

Procoagulant activity: PT was significantly elevated from the baseline by 30min after trauma (Fig 3). Administration of TXA (10mg/kg) prior to trauma (TXA-BT) significantly inhibited the elevation of PT at 30min after trauma as shown in vehicle and TXA-AT, and PT and PT% (percent change of PT to baseline) of TXA-BT were significantly lower than those of vehicle and TXA-AT. However, PT was significantly elevated at 120min from baseline in all three groups regardless of administrating TXA prior to or after trauma, and was not affected by resuscitation of FWB as shown in vehicle treated group. In contrast to Vehicle and TXA-AT showing no significant difference in PT and PT% between 30min and 120min, both PT and PT% were significantly higher at 120min than those at 30min in TXA-BT. APTT was shown to have the same pattern of change as PT, and PT%, but there was no statistical difference at 30min among the groups. The aPTT and aPTT% of TXA-BT was significantly higher at 120min than those at 30min (Fig 3). The levels of fibrinogen significantly declined at 30min after trauma and were partially restored after FWB transfusion in all three groups (Fig 3). However, no significant difference was found among the groups. These data showed that TXA given prior to trauma can postpone the development of coagulopathy during trauma and hemorrhage as shown by inhibiting the rise of PT at 30min independent of fibrinogen levels.

Plasmin(ogen)/Fibrin(ogen) ratio in traumatized liver: Immunostaining for plasmin(ogen) and fibrin(ogen) was performed at the injured site in the liver for each of the three groups (the representative images of vehicle and TXA-BT were shown at Fig 4A to 4E). The amount of plasmin(ogen) and fibrin(ogen) was analyzed by ImageJ (National Institute of Health) separately, and a ratio of plasmin(ogen)/fibrin(ogen) was calculated. We found that TXA pretreatment (TXA-BT) led to greater positive fibrin(ogen) staining in the clot (Fig 4C and 4E) as compared to vehicle (Fig 4B and 4D). The plasmin(ogen)/fibrin(ogen) ratio was significantly lower if TXA was given prior to trauma (TXA-BT group) as compared to the TXA given 45min after trauma (TXA-AT group) or vehicle (Fig 4F), suggesting that TXA pretreatment leads to greater fibrinogen deposition and less fibrinolytic potential due to reduced proportional amount of plasmin(ogen) incorporation into the clot and potentially increased clot stability at the site of injury. However, there was no significant difference of plasmin(ogen)/fibrin(ogen) ratio between Vehicle and TXA-AT, suggesting that the anti-fibrinolysis of TXA was not effective at the site of injured tissue if giving after clot formation.

Systemic fibrinolytic activity: Severe trauma led to a significant elevation in systemic plasmin activity at 30min in the groups of vehicle and TXA-AT`(Fig 4G). FWB resuscitation significantly reduced the elevation of plasmin activity at 120min compared to 30min in both groups. The similar change pattern was seen in TXA-BT, but no statistical significant was found at either 30min or 120min. There was no significant difference of circulatory plasmin activity among the groups at either 30min or 120 min. D-dimer was not significantly elevated at 30min in any of the groups, but was significantly increased at 120min in vehicle and TXA-AT despite FWB resuscitation (Fig 4H). However, TXA given prior to trauma (TXA-BT) significantly prevented this rise, and the D-dimer of TXA-BT was significantly lower than that of Vehicle and TXA-AT, suggesting potential reduction in fibrinolysis.

Acute lung injury: acute lung injury was suggested by the levels of the wet/dry weight ratio of lung tissue. We have previously shown that severe trauma and resuscitation elevates lung water content [22]. TXA given just prior to resuscitation (TXA-AT) led to a significantly lower wet/dry wet ratio as compared to that of vehicle (Fig 5). TXA given prior to trauma had no effect on the accumulation of water in the lung. There was a significant reduction of both plasmin(ogen) and CD11b stain in the lung of TXA-AT in comparison to Vehicle and TXA-BT. CD11b and plasminogen stain were found mostly co-localized, and CD11b+/plasmin(ogen)+ cells were found infiltrated into the alveoli in the lungs of the rats from both Vehicle and TXA-BT.

Platelet and RBC counts: Platelet and RBC counts significantly declined after trauma and hemorrhagic shock. Resuscitation with FWB restored platelet and RBC counts. There was no significant difference in platelet and RBC counts at any time point among the groups (Fig 6A and 6B), suggesting that TXA (given prior or after trauma) did not lead to additional acute consumption of platelets and RBCs after administration.

## Discussion

This study shows that TXA given prior to trauma in a manner similar to use in a semi-elective surgery can delay the development of coagulopathy as measured by PT, reduce systemic fibrinolysis as measured by D-dimer, and decrease fibrinolytic potential in injured tissue as measured by plasmin(ogen) and fibrin(ogen) distribution at injured site of tissue. TXA given as a single bolus either prior to trauma or at 45min after trauma (simulates prehospital time frame) has no effect on the systemic coagulopathy status at 120min after trauma with FWB resuscitation in this model. On the other hand, TXA given at 45min after trauma, but not prior to trauma attenuates the development of acute lung injury.

The in-vitro study conceptually suggested that the maximum anti-fibrinolytic effect of TXA on plasmin-induced fibrinolysis required competitive binding of TXA and plasmin(ogen) prior to clot initiation so that the plasmin(ogen)-TXA complex was not able to bind to fibrin strands to initiate fibrinolysis. Without pre-incubation of plasmin and TXA prior to initiation of clot formation, the anti-fibrinolytic effect of TXA on plasmin-induced fibrinolysis was diminished, suggesting that the degree of fibrinolysis was likely determined by the binding capacity of plasmin or plasminogen to fibrin during clot initiation. This was consistent with the in vivo study showing a reduced plasmin(ogen)/fibrin(ogen) ratio in damaged tissue of TXA-BT as compared to TXA-AT. By contrast, if TXA is given after trauma, it is not able to reverse the binding of plasmin(ogen) that has already bound to the fibrin clot to initiate fibrinolysis.

Using a rat model with ATC, we previously demonstrated that giving FWB at 60min after trauma in a limited volume (50% of the shed volume, equivalent to 2 units of whole blood given in humans during acute prehospital care) is not capable of fully restoring hemostatic function [23] as measured by PT despite the fact that FWB may be capable of preventing aggravation of ATC by supplement of coagulation factors, platelets, attenuation of tissue hypoxic damage through restoration of hemodynamics, and prevention of hemodilution due to resuscitation (S2 Fig). Administration of TXA prior to FWB is sought to improve the hemostatic outcome of FWB resuscitation to avert hyperfibrinolysis caused by massive tissue injury. In rats given a single dose of TXA prior to trauma, the fibrinolytic process in the traumatized tissue was significantly inhibited, characterized by significantly lower levels of D-dimer in the plasma and plasmin(ogen)-fibrin(ogen) ratio in sections of traumatized liver tissue as compared to vehicle or TXA given after trauma. Although the impact of TXA on fibrinogen/fibrin deposition or formation in the clot could not be accurately quantified, this study clearly suggests that fibrinogen/fibrin in the clot may be stabilized due to reduction of fibrinolysis by TXA treatment prior to trauma or surgery as used prophylactically to reduce bleeding. This suggests for the first time that the acute elevation of PT in response to trauma is independent of fibrinogen levels and may be linked to elevation of fibrin degradation products due to fibrinolysis at the level of traumatized tissue, and can be prevented by the anti-fibrinolytic agent TXA [24, 25]. However, FWB resuscitation with TXA given either early, at 45min within prehospital time frames, or immediately after trauma (S1 Fig) was not able to prevent the rise in PT at 120min, suggesting that other components of coagulation are independent of fibrinolysis involved in the development of coagulopathy after trauma [26].

The results of this study add to the body of evidence showing that prophylactic administration of TXA is beneficial for reducing blood loss during major surgical procedures [27], predominantly due to inhibition of plasminogen binding to forming blood clots, which stabilizes them by preventing local fibrinolysis. Certainly, the prophylactic administration of TXA is not feasibly applicable to trauma, but it is strongly suggested that the timing of TXA treatment is associated with reducing the risk of death due to bleeding as suggested by the CRASH-2 study [13]. Although the blood loss at injured tissue was not able to be quantified in current model, in order to achieve the greatest impact of TXA on hemostasis after trauma, TXA should be given at the point of injury as early as possible to enable TXA to act at the wound by inhibition of fibrinolysis in the clot, and reduce bleeding. The results from this study suggests that, for most patients who have already developed ATC, administration of TXA will not reverse the established systemic ATC, but rather be beneficial for stabilization of the clot at the local wound with active bleeding or receiving upcoming surgical procedure. This is consistent with the reduction of bleeding events in clinical trauma patients receiving early TXA treatment.

In this study, there was no significant difference in systemically measured plasmin activity among vehicle and TXA treated groups (TXA-BT or TXA-AT), suggesting that TXA might not necessarily alter the conversion of plasminogen to plasmin by tPA in circulating blood. The plasmin activity was significantly reduced to baseline at 120min after trauma in all three groups, which was more likely due to addition of plasmin inhibitors from FWB transfusion (e.g. alpha2-antiplasmin). However, in contrast to unchanged plasmin activity in plasma, the TXA did prevent elevation of d-dimer in the plasma, especially in the group with TXA prior to trauma, suggesting that prevention of plasminogen binding to forming clot is the principle mechanism of action of TXA. Since changes in plasma fibrinolytic indices beside D-dimer did not detect the activity of TXA, it may be misleading to depend on them to guide TXA therapy clinically.

This study explains the degree to which the molecular mechanism of TXA activity (namely prevention of plasminogen binding to forming fibrin stands) drives changes in clinically relevant coagulation parameters. In our model, we previously were not able to measure an increase in systemic fibrinolytic activity by ROTEM (S3 Fig); however, the increased systemic levels of tPA, plasmin activity and D-dimer after trauma and hemorrhage all suggested an increase in fibrinolytic activity [11]. The present study demonstrated that the fibrinolytic process initially started at the injured site immediately after trauma, prior to any change detectable in circulatory blood by ROTEM. Although it is still under debate clinically whether ROTEM or TEG should guide TXA administration [28], this study clearly suggests that fibrinolysis can be initiated in the clot at the injured site and that it is beneficial to use TXA early to attenuate clot fibrinolysis during massive tissue damaging, prior to evidence of systemic fibrinolysis as measured by ROTEM.

Our study did not identify any adverse effect of a single dose TXA administration within a very limited acute time window after trauma. There was no additional consumption of RBCs and platelets in either TXA treatment group suggesting no additional inappropriate thrombosis formation (also verified in the lung through histology, data not shown). By contrast, TXA treatment might lead to better outcomes in terms of attenuating development of acute lung injury, as characterized by a significant reduction in vascular permeability (lower wet/dry weight ratio) and leukocyte infiltration (less CD11^+^ cells) if given 45min after trauma (prior to resuscitation). We previously demonstrated that TXA reduced acute lung injury, which was associated with less vascular permeability, reduction of leukocyte and platelet infiltration, and attenuation of neutrophil activity and complement activation in the lung after trauma and hemorrhagic shock [22]. It has been demonstrated previously that there is an increase of plasminogen receptor externalization on the surface of activated monocytes after trauma and sepsis. Circulatory plasminogen binds to plasminogen receptors through lysine binding sites, and converts to plasmin by increased activity of tPA [29, 30]. Plasmin further causes tissue inflammation by promoting cellular transmembrane migration, extracellular matrix degradation [31], and damages vascular integrity and increases vascular permeability through activation of bradykinin signaling [21]. Consistently, the present study provided an evidence that CD11b^+^ leukocytes infiltrated into the lung after trauma and hemorrhagic shock were mostly plasmin(ogen) positive; and TXA given at 45min after trauma (TXA-AT) significantly reduced CD11b^+^/Plasmin(ogen)^+^ leukocytes infiltration into the lung, which was, at least in part, contributed to attenuation of acute lung injury. However, current study was not able to fully define whether the mechanism for inhibition CD11b^+^/Plasmin(ogen)^+^ leukocytes extravascular infiltration was due to the action of TXA on affecting circulatory plasminogen binding to plasminogen receptors or inhibiting plasminogen converting to plasmin by tPA on the surface of activated leukocyte after trauma followed by hemorrhagic shock. It is also interesting to notice that TXA-BT had no effect on reduction in wet/dry weight ratio and CD11b/plamin(ogen) content in the lung, which could be due to the possibility that TXA was consumed in the clot, lost during hemorrhage, or couldn’t preferably affect non-activated leukocytes prior to trauma.

Clinical studies in both civilian and military trauma suggest that early administration of TXA improves overall survival rates [14, 20] even when administered empirically, or independently of measured hemostasis status. As reported in CRASH-2 study [13], the benefit of TXA in trauma is strictly depended on the timing of administration. However, it has not been well defined in any of clinical trial whether the improvement of survivability by TXA is due to restoration of systemic hemostasis or any function independent to anti-fibrinolysis. As of now, TXA has not been FDA approved to treat trauma patients, and there was no clinical guideline for administration of TXA in trauma. The current study clearly suggested that TXA administered at prehospital time frame was not able to restore systemic ATC, but rather be beneficial to improve the outcome of resuscitation with FWB by mitigating the inflammatory response in the lung independent to anti-fibrinolysis. In parallel with other recent studies [32, 33], it will be worthwhile to investigate the anti-inflammatory effect of TXA in different animal models and clinical setting to better understand the role of TXA under the complex response to trauma. In this animal model, the fibrinolysis in the clot of damaged tissue was attenuated by TXA if administered prior to trauma, which provided a mechanistic evidence of anti-fibrinolytic effect of TXA at the site of injured tissue, which might explain why the benefit of TXA was especially seen in bleeding trauma or trauma with massive transfusion as the results of clinical trials [13, 14]. Therefore, administration of TXA is surely recommended prior to surgical procedure in acute trauma to reduce the bleeding by attenuating fibrinolysis at the wound of surgery. Certainly, the current study is not able to overcome the limitation of using a rodent model at a relatively short time window after trauma. As demonstrated previously [11, 12, 34], this rodent model recapitulated the similar pathophysiologic response including ATC as shown in severe trauma patients, however, due to the differences in species and complexity of trauma and hemorrhage pattern, the actual intensity of the response to trauma and treatment could be varied to human. Furthermore, the I.V. infusion dose of TXA for 8hr was not applied in this study as described in the CRASH-2 study, which was still unknown whether it could be necessary for sustainable effect on organ protection and hemostasis. Therefore, the optimal dosing and the effect of TXA on the outcome of both hemostasis and organ protection in trauma should be further validated in prospective randomized trials.

## Conclusions

The data show that in rats, limited FWB resuscitation restores hemodynamic function but does not completely correct ATC, and administration of TXA within simulated prehospital time frame (45min after trauma) followed by limited FWB resuscitation is not capable of correction of established systemic ATC by preventing the elevation of PT, but rather may improve overall outcomes of FWB resuscitation by attenuation of acute lung injury, as characterized by a reduction in vascular permeability and leukocytes infiltration. TXA given prior to trauma reduced levels of fibrinolysis at the site of tissue injury and circulatory d-dimer levels, and delayed development of coagulopathy independent of reduction of fibrinogen levels following trauma, which explains the effectiveness of TXA given prior to elective surgery in reducing bleeding but does not fully explain the observed clinical benefit in trauma patients. However, this study suggests that, without any other intervention, a single dose of TXA administered immediately at the point of injury is thought to be capable of inhibition of fibrinolysis in the clot, which is considered beneficial in reducing bleeding from acute surgical procedures after trauma. On the other hand, the reductions in observed biomarkers of tissue injury in this study and others suggest that, at least within one hour timeframe observed in CRASH-2 and in this rodent study, TXA may provide clinical benefit through mechanisms other than simply clot stabilization.

## Supporting information

S1 Fig PT, aPTT and fibrinogen. There was no significant different of the change in PT, aPTT and fibrinogen between the groups of Vehicle (n = 10) and TXA-AT-0min (n = 4). TXA-AT-0min: TXA was administered immediately after completion of trauma. #: significant difference compared to BL; &: significant difference compared to 30min.
(TIF)

S2 Fig Hematocrit. The hematocrit was significantly declined at 30min and restored at 120min after trauma (n = 10 per group). The hematocrit was not significant different among the groups. #: Vehicle; &: TXA-BT; $: TXA-AT. #_1_, &_1_ and $_1_: significant difference compared to baseline (BL); #_2_, &_2_ and $_2_: significant difference compared to 30min.
(TIF)

S3 Fig Lysis index by ROTEM. LI30 and LI60 were measured by ROTEM (EXTEM) at 2hr after trauma. LI30 or LI60 was not significant different among the groups of Sham (n = 3), Non-R (n = 10), and FWB (n = 10). Non-R: Trauma/hemorrhage without resuscitation; FWB: Trauma/hemorrhage with fresh whole blood resuscitation. *: Significant difference between LI60 and LI30.
(TIF)
