# Supplementary figures and images for: Effect of tranexamic acid administration on acute traumatic coagulopathy in rats with polytrauma and hemorrhage
Source: PLoS One. 2019 Oct 3;14(10):e0223406. doi: 10.1371/journal.pone.0223406 (PMC6776384; doi:10.1371/journal.pone.0223406)

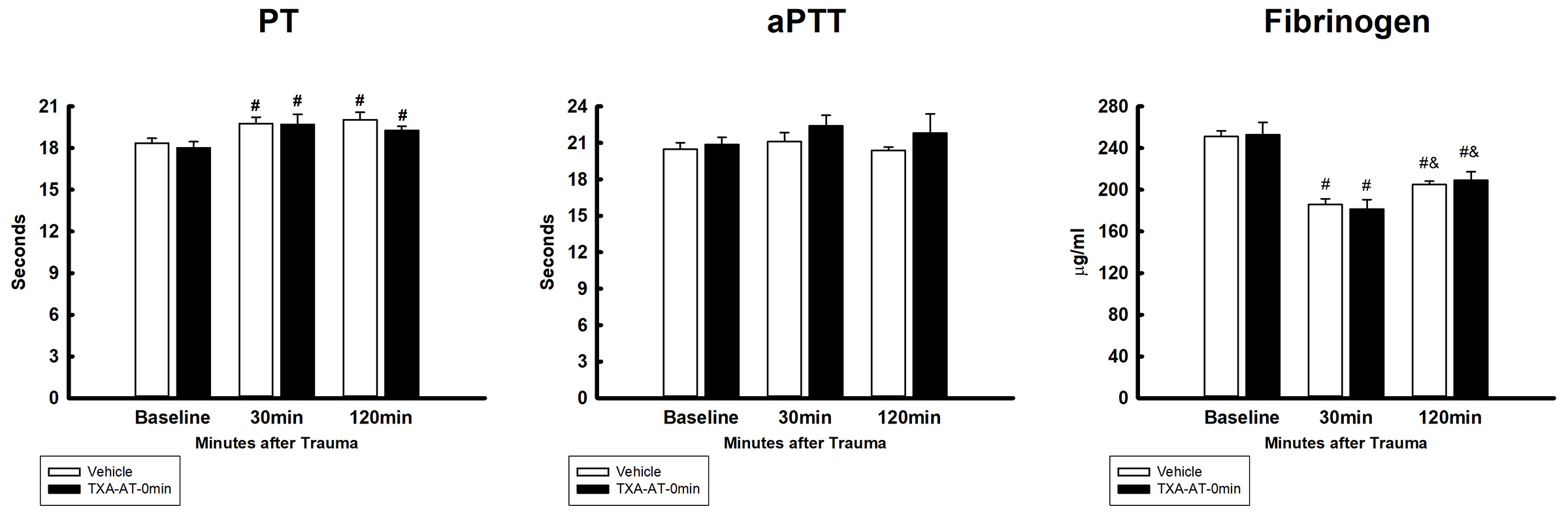

Supplement: S1 Fig — There was no significant different of the change in PT, aPTT and fibrinogen between the groups of Vehicle (n = 10) and TXA-AT-0min (n = 4). TXA-AT-0min: TXA was administered immediately after completion of trauma. #: significant difference compared to BL; &: significant difference compared to 30min. (TIF) [file pone.0223406.s001.tif]

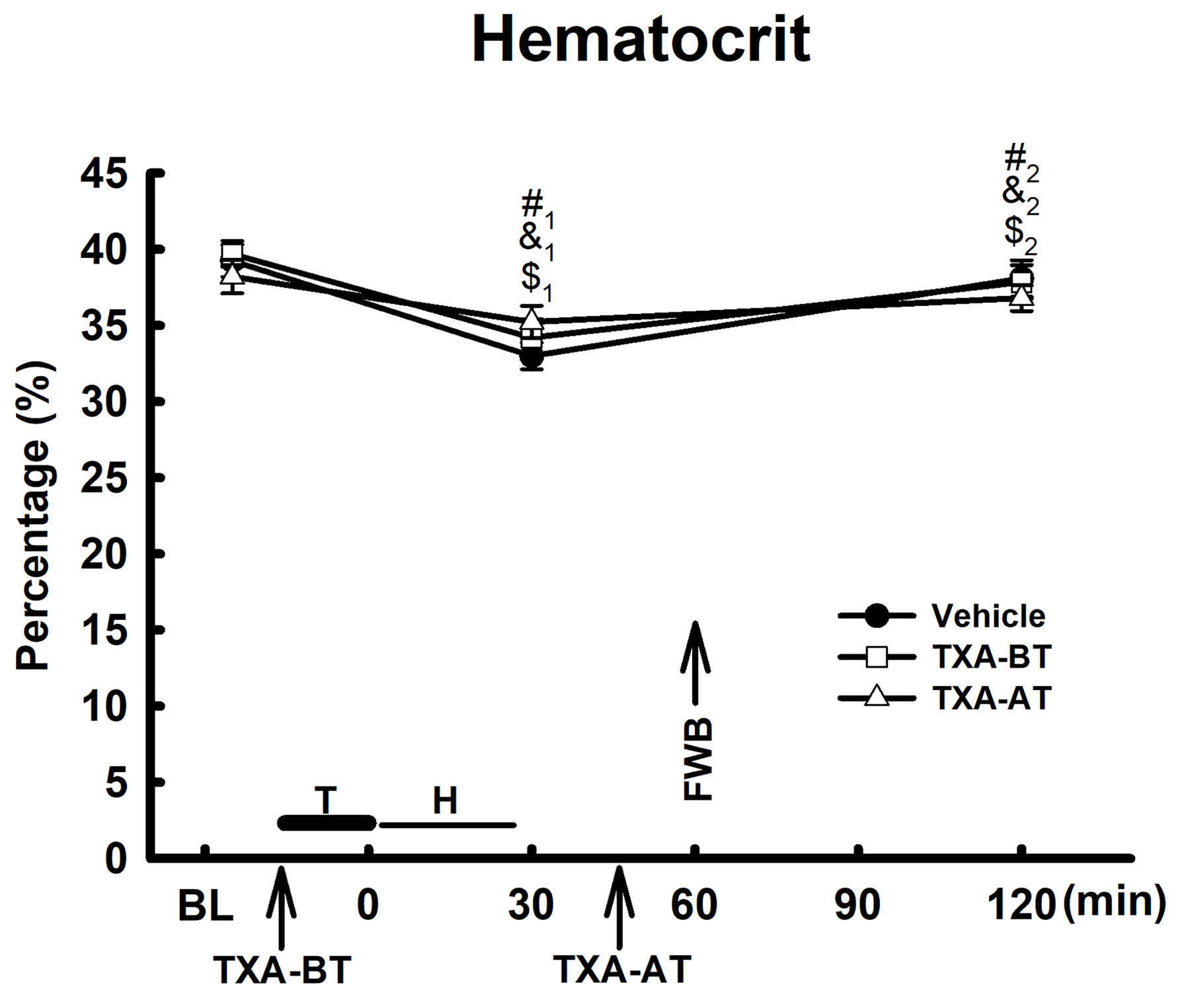

Supplement: S2 Fig — The hematocrit was significantly declined at 30min and restored at 120min after trauma (n = 10 per group). The hematocrit was not significant different among the groups. #: Vehicle; &: TXA-BT; $: TXA-AT. #1, &1 and $1: significant difference compared to baseline (BL); #2, &2 and $2: significant difference compared to 30min. (TIF) [file pone.0223406.s002.tif]

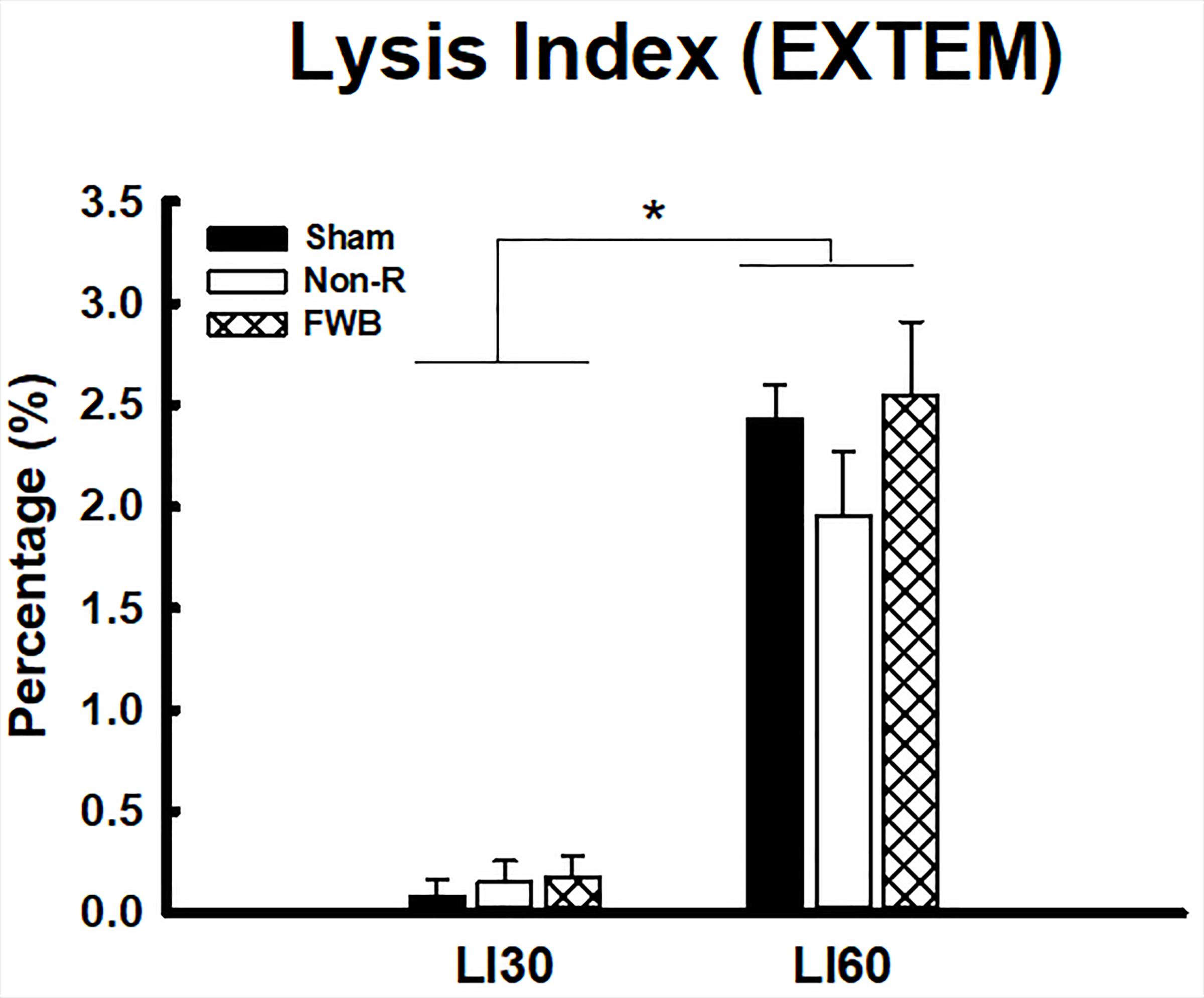

Supplement: S3 Fig — LI30 and LI60 were measured by ROTEM (EXTEM) at 2hr after trauma. LI30 or LI60 was not significant different among the groups of Sham (n = 3), Non-R (n = 10), and FWB (n = 10). Non-R: Trauma/hemorrhage without resuscitation; FWB: Trauma/hemorrhage with fresh whole blood resuscitation. *: Significant difference between LI60 and LI30. (TIF) [file pone.0223406.s003.tif]
